# Pre-treatment with the viral Toll-like receptor 3 agonist poly(I:C) modulates innate immunity and protects neutropenic mice infected intracerebrally with *Escherichia coli*

**DOI:** 10.1186/s12974-020-1700-4

**Published:** 2020-01-17

**Authors:** Sandra Ribes, Christa Arcilla, Martina Ott, Sandra Schütze, Uwe-Karsten Hanisch, Stefan Nessler, Roland Nau

**Affiliations:** 1 Institute of Neuropathology, University Medical Center Göttingen, Robert-Koch-Strasse 40, 37075 Göttingen, Germany; 2 Department of Geriatrics, Evangelisches Krankenhaus Göttingen-Weende, 37075 Göttingen, Germany

**Keywords:** Meningitis, Poly(I:C), Trained innate immunity, NK cell, Microglia, IFN-γ, CCL5/RANTES, Inflammatory response

## Abstract

**Background:**

Individuals with impaired immunity are more susceptible to infections than immunocompetent subjects. No vaccines are currently available to induce protection against *E*. *coli* meningoencephalitis. This study evaluated the potential of poly(I:C) pre-treatment to induce trained immunity. Poly(I:C) was administered as a non-specific stimulus of innate immune responses to protect immunocompetent and neutropenic wild-type mice from a subsequent challenge by the intracranial injection of *E*. *coli* K1.

**Methods:**

Three days prior to infection, mice received an intraperitoneal injection of poly(I:C) or vehicle. Kaplan-Meier survival curves were analyzed. In short-term experiments, bacterial titers and the inflammatory response were characterized in the blood, cerebellum, and spleen homogenates. NK cell subpopulations in the brain and spleen were analyzed by flow cytometry. Numbers of microglia and activation scores were evaluated by histopathology.

**Results:**

Pre-treatment with 200 μg poly(I:C) increased survival time, reduced mortality, and enhanced bacterial clearance in the blood, cerebellum, and spleen at early infection in neutropenic mice. Poly(I:C)-mediated protection correlated with an augmented number of NK cells (CD45^+^NK1.1^+^CD3^−^) and Iba-1^+^ microglial cells and a higher production of IFN-γ in the brain. In the spleen, levels of CCL5/RANTES and IFN-γ were increased and sustained in surviving poly(I:C)-treated animals for 14 days after infection. In immunocompetent animals, survival time was not significantly prolonged in poly(I:C)-treated animals although poly(I:C) priming reduced brain bacterial concentrations compared with vehicle-injected animals at early infection.

**Conclusions:**

Pre-treatment with the viral TLR3 agonist poly(I:C) modulated innate immune responses and strengthened the resistance of neutropenic mice against *E*. *coli* K1 meningoencephalitis.

## Background

Central nervous system (CNS) infections caused by *Escherichia coli* K1 strains carrying the antiphagocytic capsule K1 are common in newborns, elderly, and immunocompromised patients [1, 2]. *E*. *coli* K1 strains are also isolated from the cerebrospinal fluid (CSF) of immunocompetent adult patients after head trauma or neurosurgical procedures [3]. In immunocompromised adults, spontaneous non-traumatic community-acquired *E*. *coli* meningitis occurs with an abrupt onset and a rapid course [4, 5]. In the absence of a commercially available vaccine, CNS infections by *E*. *coli* are associated with high mortality (range 25–100%) and long-term sequelae despite available antimicrobial therapy [4, 6].

Prevention of infections in immunocompromised as well as in aged persons has proven difficult, because responses to vaccines begin to decline in healthy adults beyond 40–50 years of age [7]. To overcome this problem, vaccines incorporate adjuvants to increase sero-conversion rates in populations with reduced responsiveness [8]. Natural ligands or synthetic agonists of Toll-like receptors (TLRs) are being investigated as potential adjuvants for human vaccines [8–10]. Polyinosine–polycytidylic acid [poly(I:C)], a synthetic analog of viral double-stranded RNA (dsRNA), is recognized by the endosomal TLR3 [11]. Poly(I:C) through TLR3 recognition promotes Th1 cellular immune responses via the TLR/IL-1 receptor (TIR)-domain-containing adaptor protein-inducing IFN-β (TRIF). Poly(I:C) strongly elicited humoral and cellular immunity as part of anti-viral vaccines [12] but also enhanced the immunogenicity of the vaccine Bacille Calmette-Guérin against tuberculosis in mouse and non-human primates [13–15].

In vaccine research, several studies suggested that the exposure of the host to a certain pathogen or to single molecular patterns associated to pathogens may result in the priming of innate immune cells to fight against the target microbe but also against non-related pathogens for a relatively long period of time. By this so-called trained innate immunity, the host may acquire resistance against a broad spectrum of pathogens beyond the initial vaccine coverage [16, 17]. In the present study, the viral TLR3 agonist poly(I:C) was not used as an adjuvant, but as an inductor of heterologous (non-specific) immunity against *E. coli* K1 meningitis. Here, we demonstrated for the first time that systemic administration of poly(I:C) induced protection of immunocompromised (neutropenic) mice against one of the most common forms of Gram-negative meningitis. Modulation of innate immune responses by poly(I:C) led to an increased expression of RANTES (regulated upon activation normal T cell expressed and secreted, also called CCL5) and interferon gamma (IFN-γ), increased recruitment of natural killer (NK) cells, and higher microglial numbers and subsequently a more effective clearance of the pathogen at the local site of infection and in the systemic circulation.

## Materials and methods

### Poly(I:C)

High molecular weight poly(I:C) was purchased from InvivoGen (San Diego, CA, USA). Poly(I:C) was dissolved in 0.9% sterile saline to a concentration of 5 mg/mL and stored at − 80 °C. Poly(I:C) was administered once intraperitoneally (ip) 3 days prior infection at a dose of 2, 20, or 200 μg per mouse in a final volume of 200 μL. The control group received one single ip injection of 200 μL 0.9% NaCl (vehicle) 3 days before infection.

### Bacteria

The *E*. *coli* strain K1 (serotype O18:K1:H7) originally isolated from the CSF of a child with neonatal meningitis was used in all experimental infections [18]. Bacteria were grown over night on blood agar plates, harvested in 0.9% saline, and stored at − 80 °C. Frozen aliquots were used for the experiments and diluted with saline to the required bacterial concentration.

### Mice and monitoring

All animal experiments were approved by the Animal Care Committee of the University Medical Center Göttingen (UMG) and by the Niedersächsisches Landesamt für Verbraucherschutz und Lebensmittelsicherheit (LAVES), Braunschweig, Lower Saxony, Germany. Two to 3 months old male C57Bl/6 J wt mice bred at the Central Animal Care Facility of the UMG were used. During the experiments, animals were weighed and scored daily (0, no apparent behavioral abnormality; 1, moderate lethargy; 2, severe lethargy; 3, unable to walk; 4, dead) [19].

### Experimental design

CD11b^+^Ly-6G^+^Ly-6C^int^ neutrophils were depleted by ip injection of 50 μg of anti-Ly6G monoclonal antibody (mAb, clone 1A8, BioXcell, West Lebanon, NH) in C57Bl/6 J wt animals [18]. Anti-Ly6G mAb was administered daily starting 4 days before infection with a total of seven injections (from day − 4 to day + 2, infection performed at day 0). Neutropenic and immunocompetent animals received poly(I:C) or buffer ip 72 h prior to infection. Meningoencephalitis was induced by injection of 10 μL of a saline solution containing *E*. *coli* K1 into the superficial right frontal neocortex of anesthetized animals. In survival experiments, animals were monitored over a 14-day period after infection. In bacteriological studies, neutropenic and immunocompetent animals were sacrificed 30 h and 22 h after infection, respectively. Blood and tissue homogenates were obtained to determine bacterial titers and cytokine/chemokine levels. In additional experiments, vehicle-injected and poly(I:C)-primed neutropenic infected animals were used for flow cytometric analysis.

### Sample processing

At the end of the experiment, a blood sample was obtained in anesthetized animals by intracardiac puncture, and 1:10 dilutions of blood were plated on blood agar plates to determine bacterial concentrations (detection limit, 100 colony-forming units (CFU)/mL). Anaesthetized animals were sacrificed by cervical dislocation. Half of the spleen and half of the cerebellum were homogenized in 0.9% saline. Ten microliters of each of the homogenates were serially diluted in 0.9% saline and plated on blood-agar plates to quantify bacterial concentrations (detection limit, 200 CFU/mL and 40 CFU/mL in cerebellar and spleen homogenates, respectively). The rest of the homogenates were stored at − 20 °C until measurement of cyto- and chemokines by ELISA.

### Cyto-/chemokine measurements

Levels of CCL3 (macrophage inflammatory protein-1α, MIP-1α), CCL5/RANTES, and IFN-γ were chosen as representatives of poly(I:C)-inducible spectrum of the cyto- and chemokines [20]. Concentrations were measured in cerebellar and splenic homogenates by DuoSet ELISA Development Kits (R&D Systems, Wiesbaden, Germany) according to the manufacturer’s instructions [18]. The sensitivity was 7.5 pg/mL for all immunomodulators.

### Flow cytometry

Leukocytes were evaluated in the spleens and inoculated hemispheres of neutropenic mice pre-conditioned with 200 μg poly(I:C) (*n* = 5) or vehicle (*n* = 6) 30 h after infection by multi-color flow cytometry as previously described [10, 21]. The following antibodies were used (all from BioLegend or eBioscience): CD45 (30-F11), CD4 (RM4-5), CD27 (LG.3A10), CD11b (M1/70), Ly6C (HK1.4), CD3 (145-2C11), CD25 (PC61.5), CD19 (eBio1D3), and NK1.1 (PK136). Data were acquired on a FACS Canto™ II (BD Bioscience) device and analyzed using FlowJo software (version 8.8; Tree Star).

### Histological analysis

Paraffin-embedded, 2-μm coronal brain sections were analyzed from neutropenic animals sacrificed 30 h after infection by observers blinded to the treatments. Ionized calcium-binding adaptor molecule 1 (Iba-1) which is upregulated during microglial activation [22] was used to identify and quantify microglia. In each animal, Iba-1-positive cells were quantified in six neocortical regions and the hippocampal formation of the left brain hemisphere (total, seven regions). Microglial activation in each of the seven scored regions was assessed by a previously described cell activation score (AS) according to the most abundant morphology observed [23]. The Iba-1 staining revealed four cell morphologies according to gradual steps of microglial activation [23, 24]. For each animal, the number of Iba-1^+^ cells and the scores of the individual fields were added and then divided by the number of scored regions [18].

### Statistical analysis

Kaplan-Meier survival curves were plotted, and survival times were analyzed by the log-rank test. The Bonferroni-Holm method was used to correct for repeated testing. Differences in bacterial titers, cytokine/chemokine levels, FACS-analyzed cell subpopulations, microglial numbers, and microglial AS between poly(I:C) and buffer groups were analyzed by Mann-Whitney *U* test. Data were expressed as medians (25th/75th percentiles). The correlation between bacterial titers and cytokine/chemokine levels was analyzed using Spearman’s rank correlation coefficient *r*_s_. For all analyses, GraphPad Prism version 5 (GraphPad Software, San Diego, CA) was used. *P* < 0.05 was considered significant.

## Results

### Poly(I:C) protects neutropenic mice against *Escherichia coli* K1 meningitis in a dose-dependent manner

The majority of patients with *E*. *coli* K1 meningitis have an impaired immune system. We have shown that CD11b^+^Ly-6G^+^Ly-6C^int^ granulocytes are critical elements in the early host defense against *E*. *coli* meningitis [18]. Moreover, previous work suggested that the protective effect of TLR stimulation was stronger in neutropenic than in wt mice [10]. For this reason, a dose-finding experiment was performed in anti-Ly-6G-depleted mice (*n* = 6/group) to test the efficacy of three different doses of poly(I:C) (2, 20, and 200 μg/mouse) versus vehicle-treated animals. Mice were pre-treated with poly(I:C) or saline 3 days before induction of meningitis by intracerebral injection of 2.5 × 10^3^ CFU *E*. *coli* K1/mouse. Fourteen days after infection, only two out of six animals pre-treated with buffer or poly(I:C) 2 μg/mouse survived, while all animals pre-treated with poly (I:C) 20 or 200 μg/mouse survived. Consequently, the dose of 2 μg/mouse was discarded.

**Fig. 1:**
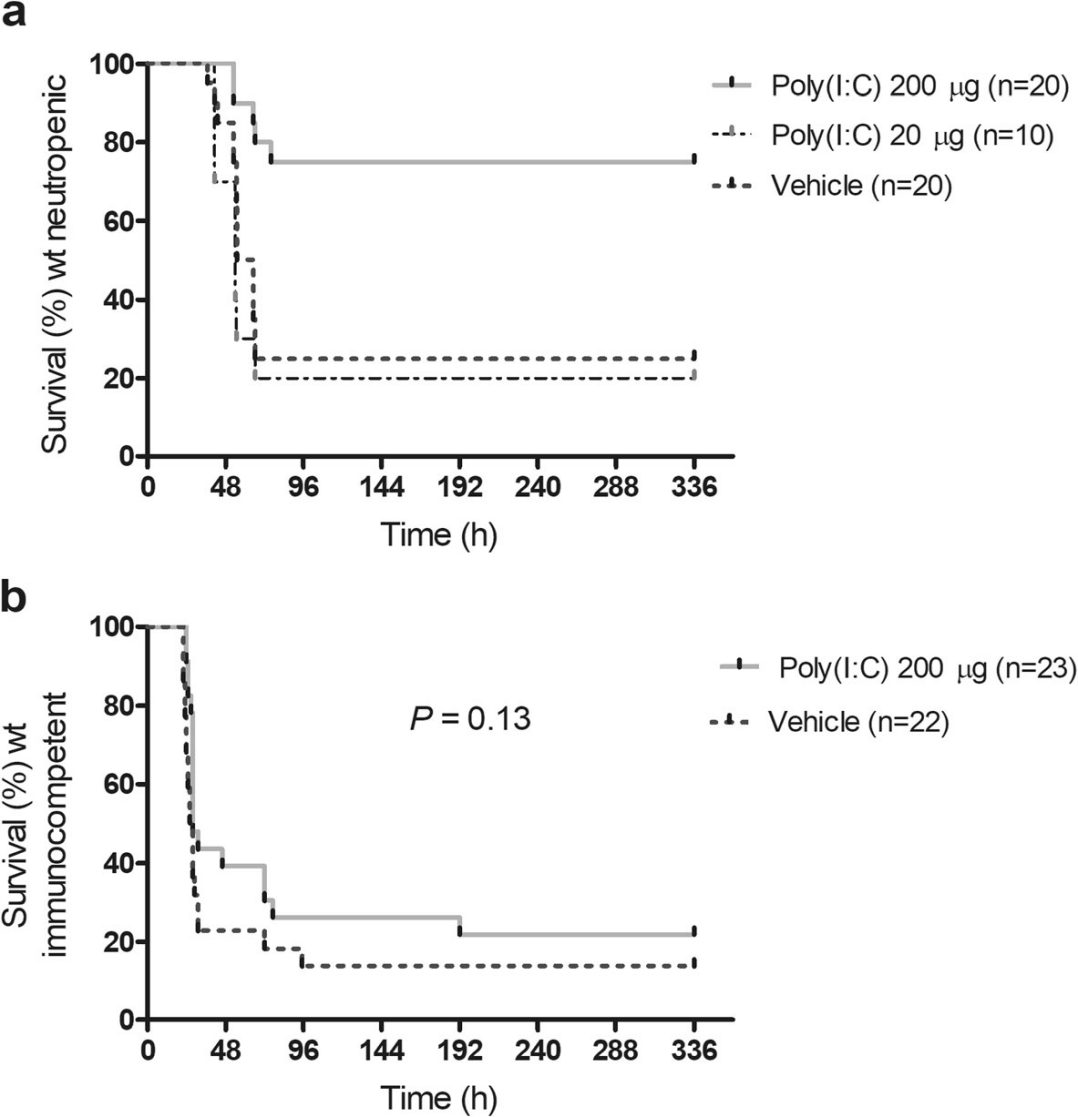
Effect of poly(I:C) pre-treatment on *Escherichia coli* meningitis in **a** neutropenic and **b** immunocompetent mice. **a** Survival time of neutropenic mice was significantly increased after pre-treatment with poly(I:C) at a dose of 200 μg compared to the vehicle group (*P* = 0.0016, log-rank test). Survival was 75% (15/20) when pre-treated with poly(I:C) 200 μg versus 25% (5/20) when receiving vehicle (*P* = 0.0038, Fisher’s exact test). Median survival time was similar in vehicle-treated mice (60 h) and in animals pre-treated with poly(I:C) 20 μg (54 h) (*P* = 0.71, log-rank test). **b** Survival time after infection was not significantly longer in wt mice pre-treated with poly(I:C) 200 μg (*P* = 0 .13, log-rank test). Survival 14 days after infection was 21.7% (5/23) in animals pre-treated with poly(I:C) 200 μg versus 13.6% (3/22) in the control group (*P* = 0.70, Fisher’s exact test)

In three further experiments, the protective effect of poly(I:C) was evaluated in neutropenic animals at a dose of 20 μg and 200 μg versus vehicle using a tenfold higher bacterial inoculum (2.5-6 × 10^4^ CFU/mouse) (Fig. 1). Survival time of neutropenic mice was significantly increased by administration of poly(I:C) 200 μg compared to the vehicle group (*P* = 0.0016, log-rank test; Fig. 1a). Survival was 75% (15/20) in pre-treated with poly(I:C) 200 μg versus 25% (5/20) when receiving vehicle (*P* = 0.0038, Fisher’s exact test). Median survival time was similar in vehicle-treated mice (60 h) and animals pre-treated with poly(I:C) 20 μg (54 h) (*P* = 0.71, log-rank test).

### Poly(I:C) has a mild effect on immunocompetent mice with *E*. *coli* K1 meningitis

Because *E*. *coli* K1 infections can also affect young healthy individuals, we evaluated the effect of poly(I:C) in mice with an intact immune system. Poly(I:C) conferred a mild protection in immunocompetent animals, which failed to reach statistical significance. Survival time after infection was not significantly prolonged in mice treated with poly(I:C) 200 μg (*P* = 0.13, log-rank test; Fig. 1b). Survival 14 days after infection was 21.7% (5/23) in animals pre-treated with poly(I:C) 200 μg versus 13.6% (3/22) in the control group (*P* = 0.70, Fisher’s exact test).

### Poly(I:C)-primed neutropenic mice display decreased bacterial concentrations in the cerebellum, spleen, and blood at the early phase of infection

**Fig. 2:**
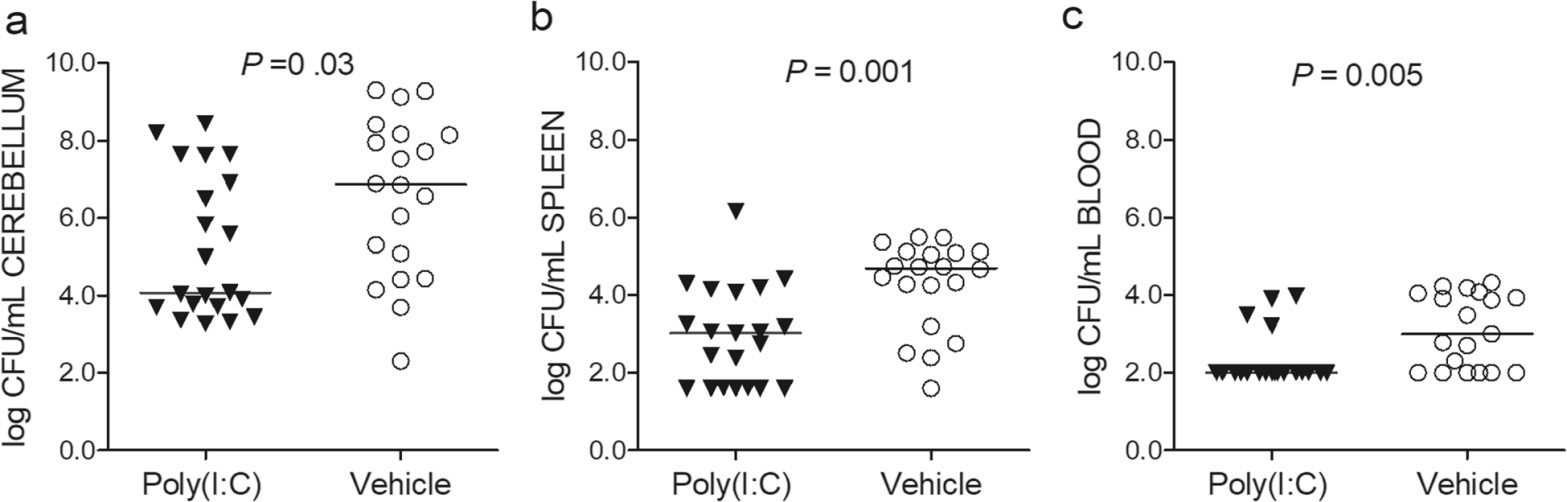
Impact of poly(I:C) on neutropenic wt animals at early infection. Bacterial concentrations were decreased in neutropenic mice (*n* = 19–21/group) pre-treated with 200 μg poly(I:C) compared with vehicle-treated animals 30 h after *E*. *coli* K1 infection in the **a** cerebellum homogenates (*P* = 0.03), **b** spleen homogenates (*P* = 0.001), and **c** blood (*P =* 0.005). Each symbol represents an individual mouse. Horizontal bars indicate median values. Statistical analysis was performed by the Mann-Whitney *U* test

We next assessed whether poly(I:C)-induced prolonged survival was associated with a more efficient inhibition of bacterial replication at the site of injection (brain) and in the systemic compartments at early infection. Therefore, bacterial loads were quantified in neutropenic wt mice sacrificed 30 h after infection in two different experiments (Fig. 2). Mice pre-treated with 200 μg poly(I:C) showed reduced bacterial titers in cerebellum homogenates compared with vehicle-treated animals (*P* = 0.03, Mann-Whitney *U* test; Fig. 2a). Bacterial concentrations in spleen homogenates and blood were significantly lower in poly(I:C)-primed animals than in the control group (*P ≤* 0.005, Mann-Whitney *U* test; Fig. 2b, c).

### Poly(I:C)-treated immunocompetent animals exhibit significantly decreased bacterial concentrations in the cerebellum at the early phase of infection

**Fig. 3:**
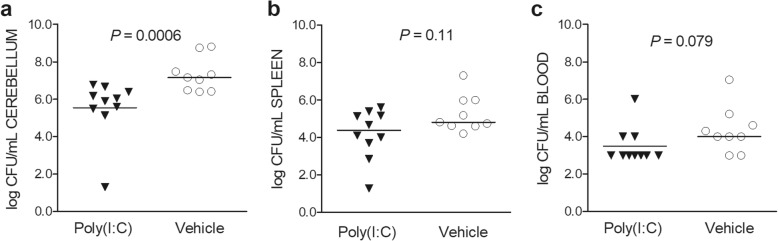
Impact of poly(I:C) on immunocompetent wt animals at early infection. Bacterial concentrations were reduced in wild-type mice (*n* = 9–10/group) pre-treated with 200 μg poly(I:C) compared with vehicle-treated animals 22 h after infection in the **a** cerebellum homogenates (*P* = 0.0006), but not in the **b** spleen homogenates (*P* = 0.11) and **c** blood (*P =* 0.079). Each symbol represents an individual mouse. Horizontal bars indicate median values. Statistical analysis was performed by the Mann-Whitney *U* test

We next evaluated the effect of poly(I:C) pre-treatment in infected immunocompetent animals. In two independent experiments, poly(I:C)-primed immunocompetent animals sacrificed 22 h after infection showed lower bacterial titers in cerebellum homogenates than vehicle-treated animals (*P* = 0.0006, Mann-Whitney *U* test, Fig. 3a). Bacterial loads in the spleen homogenates and blood tended to be reduced in poly(I:C) pre-treated animals than in control animals (*P* = 0.11, and *P* = 0.079, respectively, Mann-Whitney *U* test; Fig. 3b, c). Blood bacterial cultures were below the level of detection in 7/10 poly(I:C)-treated animals and in 2/9 vehicle-injected controls (*P* = 0.070, Fisher’s exact test).

### Poly(I:C)-treated neutropenic animals show a higher density of microglial cells and a lower microglial AS at the early infection

**Fig. 4:**
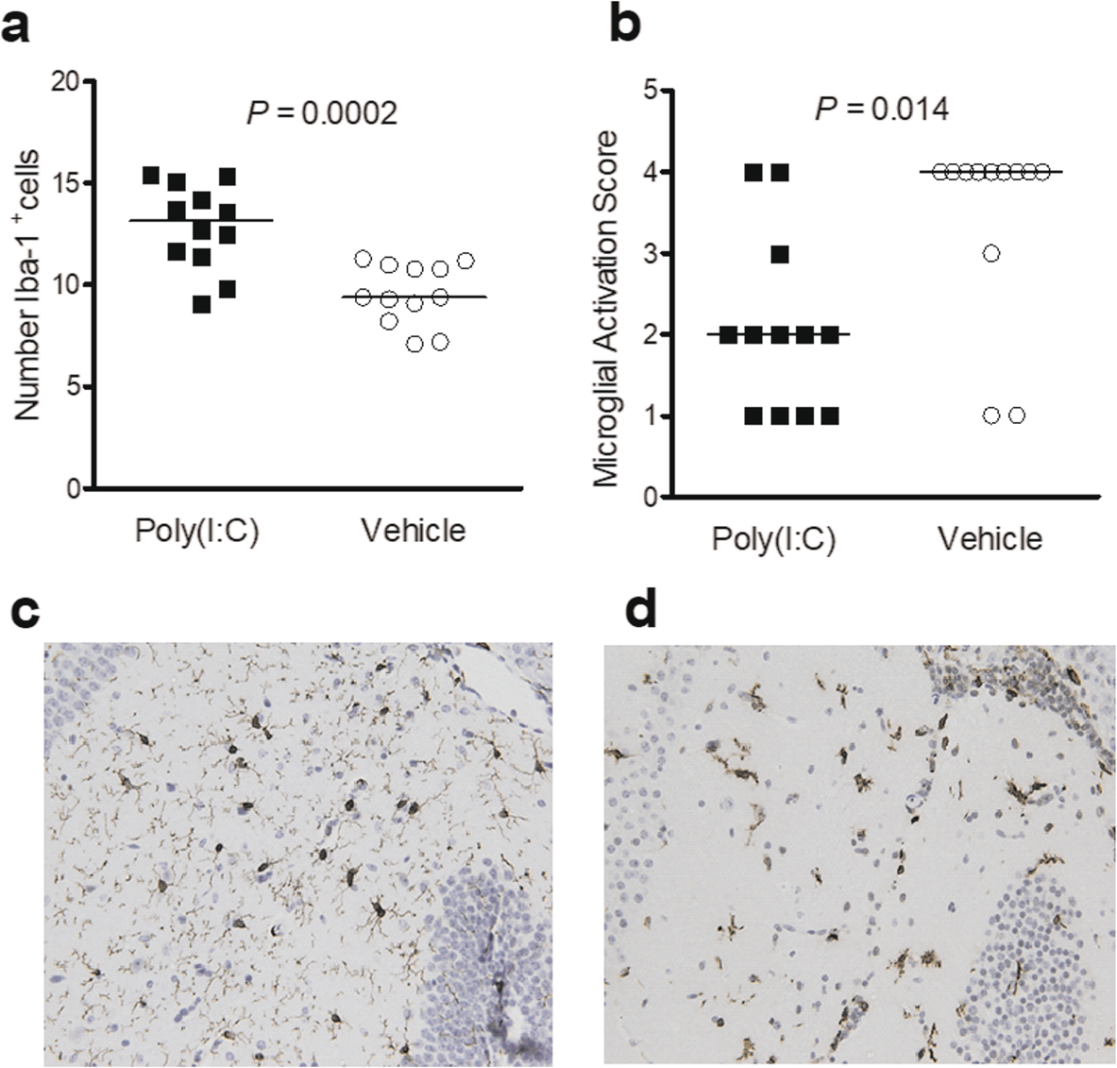
Microglial cells in infected poly(I:C)-pre-treated and control mice. **a** The number of Iba-1^+^ microglial cells in infected animals primed with 200 μg poly(I:C) was increased compared with the control group (*P* = 0.0002, Mann-Whitney *U* test). **b** A microglial activation score (AS) of 1 was given when cells had relative big somata and fine ramifications, an AS of 2 was given to hyperthrophic cells with thicker branches, while AS3 and AS4 were assigned to bushy and ameboid cells [24, 25]. The AS was higher in vehicle-treated compared with poly(I:C)-pre-conditioned mice (*P* = 0.01, Mann-Whitney *U* test). Each symbol represents an individual mouse. Bars indicate median values. **c** Poly(I:C)-pre-treated neutropenic animals mostly exhibited microglia with a hypertrophic-bushy morphology [median AS (25./75. percentile), 2.0 (1.00/2.75)], while **d** in the vehicle-injected animals, microglia more often showed an ameboid appearance [AS 4.00 (3.25/4)]

Iba-1-stained brain sections served to quantify microglial densities and activation in neutropenic mice sacrificed 30 h after *E*. *coli* K1 injection (*n* = 12/group). The number of Iba-1^+^ cells in infected animals that received 200 μg poly(I:C) was significantly increased compared with the control group (Fig. 4a, *P* = 0.0002, Mann-Whitney *U* test, *n* = 12/group). Conversely, the microglial AS was higher in vehicle-treated compared with poly(I:C)-pre-conditioned mice (Fig. 4b, *P* = 0.01, Mann-Whitney *U* test). Poly(I:C)-treated neutropenic animals mostly exhibited cells with a hypertrophic-bushy morphology [Fig. 4c, median AS (25./75. percentile), 2.0 (1.00/2.75)], while microglia in vehicle-injected animals more often showed an ameboid appearance [Fig. 4d, 4.00 (3.25/4)]. Microglial AS strongly correlated with *E*. *coli* K1 concentrations in the brain (*r*_s_ = 0.63, *P* = 0.0011; *n* = 24).

### Poly(I:C) induces the recruitment of NK cells (CD45^+^NK1.1^+^CD3^−^) into the brain and higher production of IFN-γ in neutropenic infected mice

**Fig. 5:**
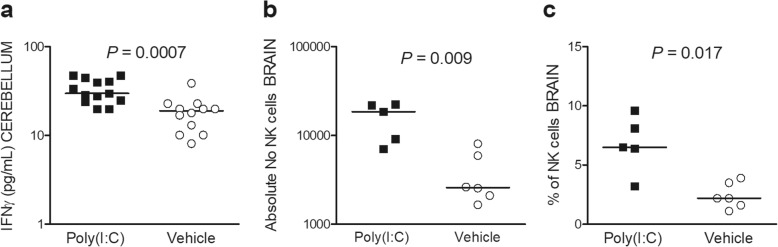
Brain IFN-γ levels and NK cell numbers 30 h after *E*. *coli* infection in neutropenic mice after poly(I:C) pre-conditioning. **a** IFN-γ levels were higher in poly(I:C)-treated than in control animals (*P* = 0.0007). **b** Poly(I:C) pre-treated infected animals exhibited higher absolute numbers of NK cells in the brain compared with the vehicle group (*P* = 0.009). **c** The percentage of NK cells among the total amount of CD45^+^ leukocytes was significantly increased in the brain of poly(I:C)-primed animals compared with the vehicle group (*P* = 0.017). Each symbol represents an individual mouse. Horizontal bars indicate median values. Statistical analysis was performed by the Mann-Whitney *U* test

During acute infection, cerebellar IFN-γ levels were significantly higher in poly(I:C)-treated neutropenic mice than in control animals (*P* = 0.0007, Mann-Whitney *U* test; Fig. 5a). Since NK cells might be relevant early producers of IFN-γ in acutely infected mice [25], we quantified NK cell numbers (CD45^high^ CD3^−^ NK1.1^+^) by flow cytometry. The percentage of NK cells among the total amount of CD45^+^ leukocytes was significantly increased in the brain of poly(I:C)-primed animals compared to vehicle-pre-treated animals (*P* = 0.017, Mann-Whitney *U* test; Fig. 5c). In line, poly(I:C) pre-treated infected animals exhibited also higher absolute numbers of NK cells in the brain compared to the vehicle group (*P* = 0.009, Mann-Whitney *U* test; Fig. 5b).

In the cerebellum, pre-conditioned neutropenic animals also showed reduced levels of CCL3 [7.4 (7.4/917.2) pg/mL] compared with the control animals [655.4 (116.3/2589) pg/mL; *P* = 0.09, Mann-Whitney *U* test]; decreased levels of CCL3 significantly correlated with low bacterial loads (*r*_S_ = 0.90, *P* < 0.0001, *n* = 24).

### Effect of poly(I:C)-treatment on the production of IFN-γ and CCL5/RANTES and NK cell numbers in the spleen

**Fig. 6:**
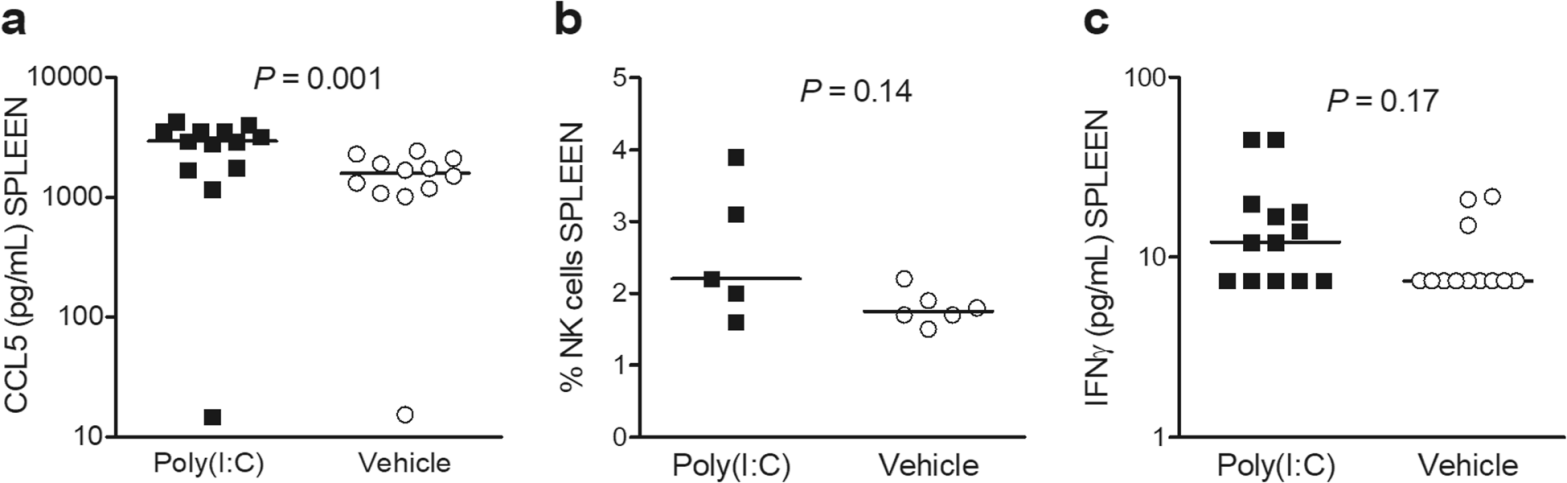
Poly(I:C)-driven innate immune responses in spleen of infected neutropenic mice. **a** CCL5 levels were significantly increased in spleen compared to vehicle-injected animals (*P* = 0.001). **b** Flow cytometry experiments showed no significant differences in the frequency of splenic NK cells. **c** There were no significant differences in the levels of IFN-γ between poly(I:C)-treated and control mice (*P* = 0.17). Each symbol represents an individual mouse. Horizontal bars indicate median values. Statistical analysis was performed by the Mann-Whitney *U* test

Poly(I:C)-primed neutropenic animals exhibited significantly higher levels of CCL5 in the spleen compared with vehicle-administered animals (*P* = 0.001, Mann-Whitney *U* test; Fig. 6a). CCL5 induces the proliferation and activation of NK cells. Poly(I:C)-treated animals showed slightly increased concentrations of IFN-γ (*P* = 0.10, Mann-Whitney *U* test; Fig. 6b) compared to vehicle-treated animals. Overall, reduced *E*. *coli* K1 concentrations tended to correlate with increased levels of CCL5 (*r*_S_ = − 0.34, *P* = 0.11, *n* = 24) and IFN-γ (*r*_s_ = − 0.37, *P* = 0.07, *n* = 25).

### IFN-γ and CCL5 levels remained consistently high in surviving neutropenic animals

**Fig. 7:**
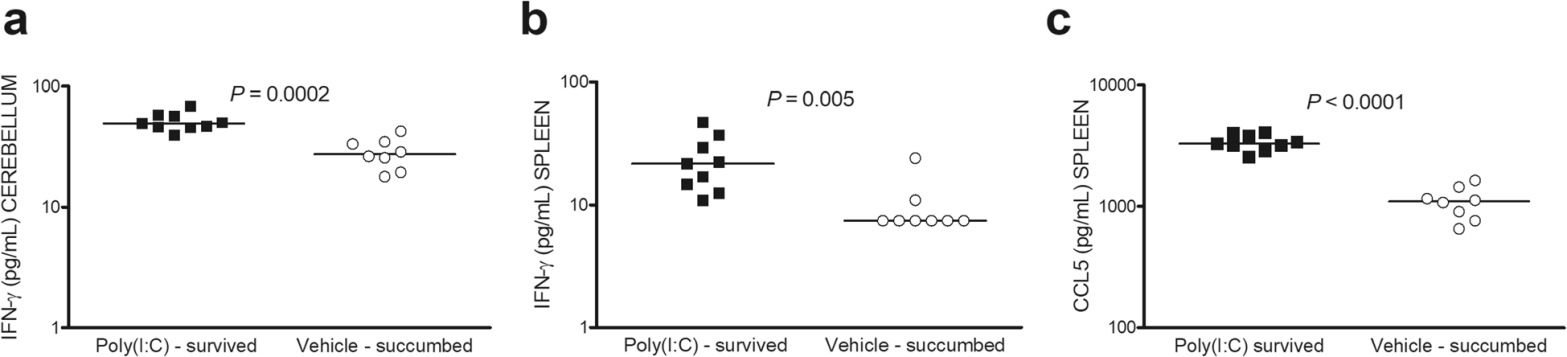
IFN-γ and CCL5 levels remained consistently high in surviving neutropenic animals. Poly(I:C)-pre-treated animals surviving the infection showed significantly higher levels of IFN-γ in the **a** cerebellum and **b** spleen, and of **c** CCL5 in the spleen, compared to controls (*P* ≤ 0.005). Each symbol represents an individual mouse. Horizontal bars indicate median values. Statistical analysis was performed by the Mann-Whitney *U* test

To assess whether increased levels of IFN-γ and CCL5 remained stable, we measured the concentrations in neutropenic animals at the time of sacrifice during a survival experiment (*n* = 10/group). Nine out of ten animals pre-treated with 200 μg poly(I:C) and 2/10 vehicle-treated animals survived. To allow statistical analysis of data, we compared the concentrations of IFN-γ and CCL5 in poly(I:C)-primed surviving animals (*n* = 9) versus vehicle-administered animals which succumbed to infection (*n* = 8) (Fig. 7). All evaluated animals succumbed to infection 41.5 h to 66 h after infection. The animals which survived the infection were sacrificed 336 h after infection. IFN-γ levels were higher in the cerebellum and spleen of poly(I:C)-treated animals surviving the infection compared to controls (*P* ≤ 0.005, Mann-Whitney *U* test). The only poly(I:C)-treated animal which succumbed to infection had an IFN-γ level of 38 pg/mL in the cerebellum, and the IFN-γ concentration was below the level of detection in the spleen. CCL5 concentrations remained sustainably increased in the spleen of surviving animals primed with poly(I:C) compared to controls (*P* < 0.0001, Mann-Whitney *U* test). The only poly(I:C)-treated animal which succumbed to infection had a CCL5 concentration in the spleen of 1522 pg/mL.

## Discussion

One of the biggest challenges in finding new strategies in the management and prevention of bacterial meningitis is to identify potent compounds that strengthen the innate immune response without exacerbating inflammation and aggravating neuronal injury. The search for enhancers of innate immunity is most relevant in meningitis forms for which conventional vaccines are not available such as *E*. *coli* meningitis.

In healthy individuals, activation of parenchymal microglia is one of the early mechanisms of the CNS to impede the entry and spread of pathogens and prevent CNS infections [26]. However, an excessive or persistent activation of microglia increases the production of neurotoxic pro-inflammatory mediators contributing to the development of neurological sequelae in meningitis patients [27, 28].

Microglia express innate immune receptors such as pattern recognition receptors including TLR3 [29]. The signaling of poly(I:C) as a viral TLR is primarily dependent on TLR3, and poly(I:C) strongly drives cell-mediated immunity and an IFN type 1 and 2 responses. Poly(I:C) has been tested as an adjuvant to several vaccines [30]. Stimulation of primary microglial cell cultures with poly(I:C) enhanced phagocytosis and intracellular killing of *E*. *coli* K1 without inducing a strong concomitant release of pro-inflammatory cytokines and chemokines [31]. In the present study, we investigated the potential of poly(I:C) as heterologous stimulator of microglia and other innate immune cells to confer protection in immunocompetent and immunosuppressed mice against *E. coli* K1 meningitis. We chose to use both animal models, as *Escherichia coli* K1 brain infections are clinically relevant in immunocompromised individuals and—less frequently—also in healthy adults.

Mice primed with the heterologous viral TLR agonist poly(I:C) showed increased resistance against *E*. *coli* K1 intracerebral infection. This effect was strong in neutropenic mice, whereas in immunocompetent young adult mice, the effect of poly(I:C) pre-treatment on increase of the median survival time after infection failed to reach statistical significance. This suggests that poly(I:C) administration is unable to substantially increase the resistance against *E*. *coli* infection beyond the infection resistance of a normal young individual. In neutropenic animals, the protective effect conferred by poly(I:C) was associated with a more effective bacterial clearance at the local site of infection and a lower spread of bacteria into blood circulation. During infection, poly(I:C)-pre-treated neutropenic animals showed higher recruitment of NK cells into the brain with associated increased IFN-γ production than control mice. Whether poly(I:C) is protective against infections probably depends on the dose and the interval between priming and infection: in a peritonitis model in immunocompetent mice after cecal ligation, poly(I:C) priming with a dose of 50 μg 12 h prior to surgery was not protective [32]. Poly(I:C) administration appears to be safe concerning the induction of autoimmune diseases: mice dosed i.p. with poly(I:C) every other day for 3 weeks or three times intramuscularly together with anthrax antigen twice at an interval of 2 weeks did not develop additional autoimmune symptoms [33].

IFN-γ release leads to an increase of indoleamine 2,3-dioxygenase activity, a defense mechanism against many extracellular bacteria including multi-resistant strains [34]. In *Cryptosporidium parvum* infection, after administration of poly(I:C), IFN-γ-deficient neonatal mice displayed a parasite load similar to untreated neonatal mice, suggesting that this cytokine was critical for the protection induced by poly(I:C). In addition to IFN-γ, IL-12p40 and type 1 IFNs were required for poly(I:C)-induced protection [35]. In murine *Yersinia enterocolitica* infection, systemic administration of poly(I:C) activated NK cells in the mesenteric lymph nodes and induced their IFN-γ expression. Poly(I:C)-induced NK cell activation was mediated by type 1 IFNs and IL-12p40 [36]. In the present study, unlike other pro-inflammatory compounds, IFN-γ concentrations were not positively correlated with bacterial densities (spleen: *r*_s_ = − 0.37, *P* = 0.07), and IFN-γ concentrations in the spleen and cerebellum remained high in surviving poly(I:C)-treated mice. This strongly suggests that IFN-γ release was not triggered by bacterial products, but probably represents a true consequence of stimulation of the immune system by poly(I:C). Since NK cells are the main source of IFN-γ during an acute infection, our finding suggests an involvement of NK cells in the protective action of poly(I:C) observed in this study. NK cells make up to 15% of all peripheral blood lymphocytes [37]. NK cells participate in many immunological and regulatory processes including viral, bacterial, and fungal infections [38]. In mice, intraperitoneal injection of *Streptococcus* group B and *Streptococcus suis* rapidly induced IFN-γ release and NK cells were the major cell type responsible for its production during the acute phase of the infection [39].

CCL5/RANTES contributes to the recruitment of granulocytes into inflammatory sites [40]. CCL5 concentrations in the spleen of infected animals were increased after poly(I:C) pre-stimulation, and—as IFN-γ levels—CCL5 concentrations showed a tendency to inversely correlate with bacterial concentrations. The effect of poly(I:C) administration on CCL5 release is immediate. As early as 24 h after the intraperitoneal injection of poly(I:C) 200 μg, CCL5 levels in serum of non-infected immunocompetent wt mice were significantly higher compared to buffer-treated animals (data not shown). CCL5 concentrations in serum remained significantly increased at the time of the intracerebral challenge with *E*. *coli* K1 (69 h after primining with poly(I:C)). Together, these observations suggest a potential contribution of lymphocytes, CCL5, and IFN-γ in the protective effect of poly(I:C) pre-stimulation.

We previously demonstrated that upon TLR stimulation primary cultures of microglial cells transform into a rounded “amoeboid” morphology, release pro-inflammatory cytokines and nitric oxide, and develop enhanced phagocytosis and intracellular killing of *E*. *coli* K1 compared to unstimulated cells [31, 41, 42]. Thus, in the presence of high amounts of bacteria, microglial cells acquire an amoeboid morphology with a fully functional phagocytic status. Whether phagocytic microglia also produce a specific pattern profile of cytokines is not easy to determine due to the high heterogeneity of microglial subpopulations and to the rapidity by which these cells can respond to stimuli [43]. In poly(I:C)-pre-treated infected mice, the density of microglial cells in the cerebral cortex was higher and the activation score lower than in infected control mice. We hypothesize that the higher density of microglia contributing to the increased resistance to infections was a consequence of poly(I:C) pre-stimulation, whereas the higher microglial AS in infected control mice probably was caused by the presence of high bacterial loads.

CCL3/MIP-1α is produced by activated microglia and induced recruitment of various inflammatory cells into sites of inflammation [44]. At early infection, CCL3 cerebellar concentrations of poly(I:C)-pre-treated mice were lower than those in control mice. In vivo, CCL3 concentrations correlated with bacterial loads. This suggests that the influence of bacterial pro-inflammatory products on cerebellar CCL3 concentrations was greater than the action of poly(I:C).

## Conclusions

The present study supports the validity of the concept of trained innate immunity. The “viral” TLR agonist poly(I:C) confers protection against a bacterial infection with a Gram-negative pathogen. Poly(I:C) is not only effective as an adjuvant to viral and bacterial vaccines, but has an immunoprotective activity itself against *E*. *coli* K1 meningitis in the immunocompromised host. This makes poly(I:C) a promising candidate for the induction of trained innate immunity in immunocompromised patients with a high risk of infections. Before initiation of a clinical study in immunocompromised patients at a high risk of infections, further pre-clinical studies should assess how long the effect of a single injection of poly(I:C) will last, whether repeated poly(I:C) injections will be more effective than a single dose, and whether this approach will be effective with other bacteria than *E*. *coli*.

## Data Availability

The datasets used and/or analyzed during the current study are available from the corresponding author on reasonable request.

## Abbreviations

AS: Activation score
CFU: Colony-forming units
CNS: Central nervous system
CSF: Cerebrospinal fluid
Iba-1: Ionized calcium-binding adaptor molecule 1
IFN-γ: Interferon gamma
ip: Intraperitoneally
mAb: Monoclonal antibody
MIP-1α/CCL3: Macrophage inflammatory protein-1α
NK cells: Natural killer cells
Poly(I:C): Polyinosine–polycytidylic acid
RANTES/CCL5: Regulated upon activation normal T cell expressed and secreted
r_s_: Spearman’s rank correlation coefficient
TLR: Toll-like receptors
TRIF: TLR/IL-1 receptor (TIR)-domain-containing adaptor protein-inducing IFN-β
UMG: University Medical Center Göttingen
